# Women’s decision-making power in a context of free reproductive healthcare and family planning in rural Burkina Faso

**DOI:** 10.1186/s12905-021-01411-4

**Published:** 2021-07-22

**Authors:** Camille Beaujoin, Alice Bila, Frank Bicaba, Véronique Plouffe, Abel Bicaba, Thomas Druetz

**Affiliations:** 1 School of Public Health, University of Montreal, Montreal, QC Canada; 2 Société d’Études et de Recherche en Santé Publique (SERSAP), Ouagadougou, Burkina Faso; 3 Independent Consultant, Montreal, QC Canada; 4 Centre de Recherche en Santé Publique (CReSP), Montreal, QC Canada; 5 Center for Applied Malaria Research and Evaluation, Department of Tropical Medicine, Tulane University, New Orleans, LA USA

**Keywords:** Burkina Faso, Decision-making power, Family planning, National user fee exemption policy, Reproductive health

## Abstract

**Background:**

In 2016, the national user fee exemption policy for women and children under five was introduced in Burkina Faso. It covers most reproductive healthcare services for women including prenatal care, delivery, and postnatal care. In subsequent years, the policy was gradually extended to include family planning. While studies have shown that user fee abolition policies increase visits to health centers and improve access to reproductive healthcare and family planning, there are also indications that other barriers remain, notably women’s lack of decision-making power. The objective of the study is to investigate women’s decision-making power regarding access to reproductive health and family planning in a context of free healthcare in rural Burkina Faso.

**Methods:**

A descriptive qualitative study was carried out in rural areas of the Cascades and Center-West regions. Qualitative data were collected using individual semi-structured interviews (n = 20 participants) and focus groups (n = 15 participants) with Burkinabe women of childbearing age, their husbands, and key informants in the community. Data was analyzed using thematic analysis.

**Results:**

A conceptual framework describing women’s participation in the decision-making process was built from the analysis. Results show that the user fee exemption policy contributes to improving access to reproductive care and family planning by facilitating the negotiation processes between women and their families within households. However, social norms and gender inequalities still limit women’s decision-making power.

**Conclusion:**

In light of these results, courses of action that go beyond the user fee exemption policy should be considered to improve women’s decision-making power in matters of health, particularly with regard to family planning. Interventions that involve men and community members may be necessary to challenge the social norms, which act as determinants of women’s health and empowerment.

**Supplementary Information:**

The online version contains supplementary material available at 10.1186/s12905-021-01411-4.

## Background

According to the United Nations (UN), its Millennium Development Goals planned for 2015 regarding maternal health have not been achieved, despite a 49% decrease in maternal mortality between 1990 and 2015 in sub-Saharan Africa [1]. The Sustainable Development Goals, which were adopted after the Millennium Development Goals, aim to reduce maternal mortality to fewer than 70 deaths per 100,000 live births in sub-Saharan Africa by 2030 and to ensure access to sexual and reproductive healthcare for all women [2]. The World Health Organization (WHO) argues that most maternal deaths could be prevented by ensuring that women have access to reproductive healthcare, including prenatal care, postnatal care, and delivery [3]. Furthermore, according to WHO, access to family planning and contraception would also reduce the health risks related to unwanted pregnancies for women [3]. Therefore, WHO recommends facilitating access to reproductive healthcare and family planning, which is in line with the improvement of Universal Health Coverage, the main strategy to achieve the Sustainable Development Goals.

In order to improve access to reproductive care, the government of Burkina Faso implemented a national user fee exemption policy for women and children under five years of age in June 2016 [4]. The policy provides free screening for breast and cervical cancer, prenatal and postnatal care, deliveries and caesarean sections, universal healthcare for children under five [5], as well as fuel for in-country medical evacuations, including emergency obstetric care [6]. In many settings, abolishing user fees for pregnant women has shown positive outcomes, including an increase in women’s visits to healthcare facilities, especially among the poorest women and women from rural areas [7] as well as an increase in skilled attendant-assisted deliveries [8]. In June 2018, this user fee exemption policy was extended to include family planning in two pilot regions of Burkina Faso [9]. This extended exemption covers the cost for family planning consultations and counseling, for contraceptives (mainly injectable, implants, copper intrauterine devices, emergency contraceptive pills and condoms) and consumables, and for all related medical procedures, tests and examinations.

Despite the undeniable benefits of free healthcare policies, the problem of women’s access to reproductive healthcare remains even in the absence of user fees, particularly in rural areas. In Burkina Faso, the most recent available data suggest that only 60% of women living in rural areas gave birth in a public or private health center, versus 93% in urban areas [10]. Regarding family planning, in 2018, 23% of married women in Burkina Faso who did not want to become pregnant were not using any contraceptive method [11]. Barriers other than cost may continue to limit women’s access to healthcare. Studies conducted in Burkina Faso and other Sub-Saharan African countries have suggested the influence of many other factors, including lack of transportation, distance from health centers, poor provider-patient relationship, lack of respectful maternity care, or sociocultural practices [12–16].

One factor recently highlighted in the literature is women’s lack of decision-making power in the household regarding healthcare-seeking practices. Studies suggest that the decision to seek reproductive healthcare or to use family planning services usually rests with husbands or other members of the family [17]. The removal of user fees at healthcare facilities seems to have little or no impact on women’s decision-making power; at best, it may alleviate the need for women to negotiate the necessary financial resources with their husbands [18]. However, as identified in a recent literature review, few studies have investigated women’s lack of autonomy in decision-making (and its influence on access to healthcare) after user fee removal in sub-Saharan African countries [19]. Regarding family planning services specifically, women’s decision-making has yet to be examined in a context where these services are provided free of charge.

The aim of this study is to describe women’s decision-making power regarding access to reproductive healthcare and family planning in rural Burkina Faso. It is hypothesized that, even in a context of a national user fee exemption policy, women’s lack of autonomy continues to act as a barrier. By providing evidence about this potential barrier, the intention of this study is to provide useful information for program planners and to help define complementary interventions to further improve women’s access to reproductive care. To the best of our knowledge, this is the first study to pursue this objective in a setting where reproductive care and family planning services are provided free of charge. Moreover, this originality of this study also consists in drawing a comprehensive portrait of women’s decision-making power by cross-referencing women’s points of view with those of their husbands and community members.

## Methods

A cross-sectional qualitative study was carried out in rural Burkina Faso in January 2020, three and a half years after the introduction of the national user fee exemption policy (for children under five years and reproductive healthcare services) and about six months after the extension of this policy to include family planning services. A COREQ checklist (consolidated criteria for reporting qualitative research) was used to ensure the methodology of the study is correctly reported [20].

### Study site

**Fig. 1:**
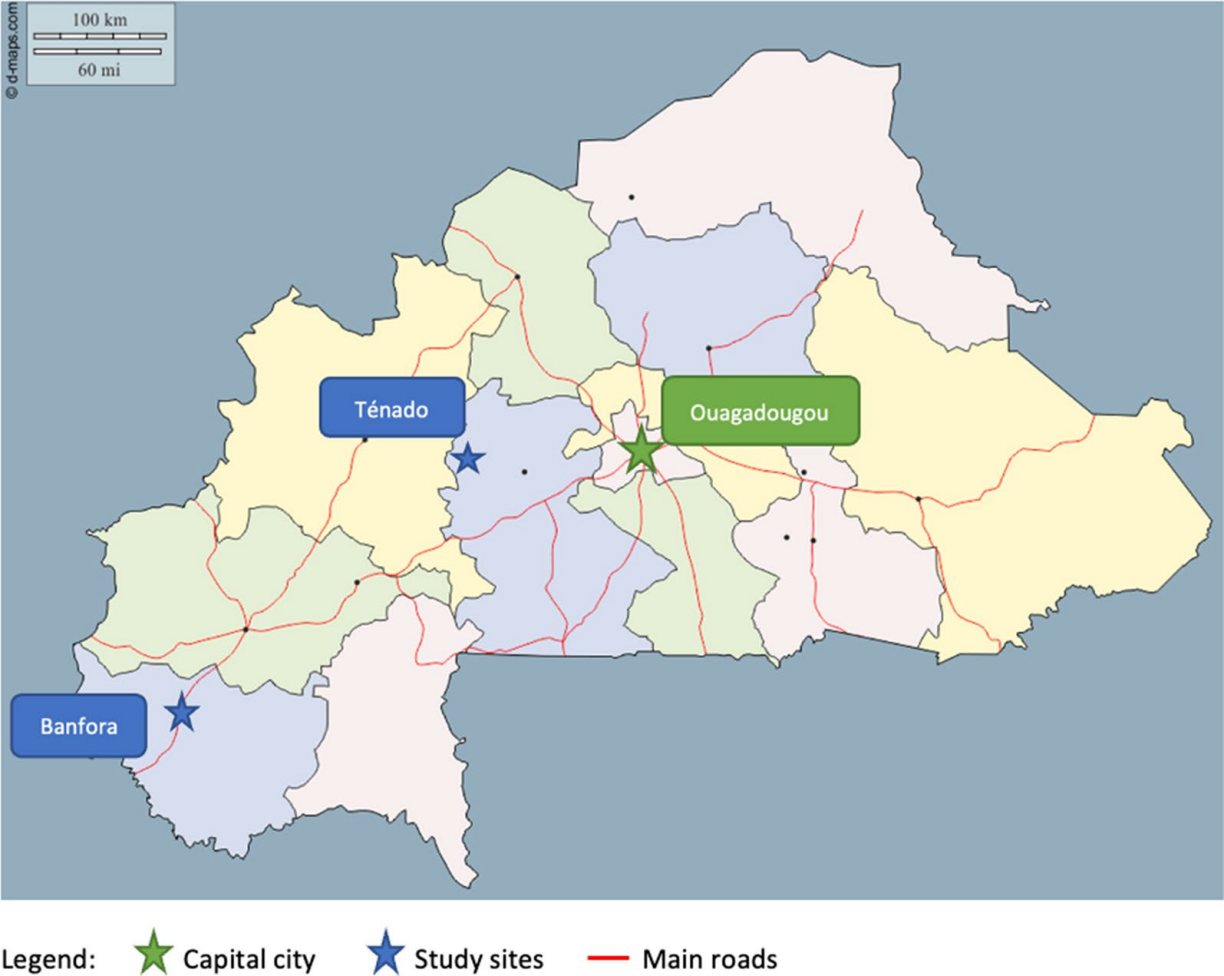
Study sites in Burkina Faso (adapted from https://www.d-maps.com with Microsoft Word, version 16.49) [47]

Data collection took place in two health districts of Burkina Faso. These districts were sampled because i) they are located in a safe area, where no terrorist attacks have been recorded within the two years preceding the study, and ii) they are located in the two pilot regions where the user fee exemption policy for family planning services was implemented, namely the Cascades and the Center-West regions. These districts were Ténado (185,873 inhabitants in 2017) and Banfora (392,498 inhabitants in 2017) [21]. They are respectively located 130 km and 440 km from Ouagadougou, the capital of Burkina Faso, and Banfora is located 85 km from Bobo-Dioulasso, the second largest city in the country (see Fig. 1). For each health district, two sites were selected with the assistance of the district health authorities based on three additional criteria: i) they were distant from large cities (the problem of access to reproductive care and family planning being particularly salient in rural areas), ii) a Centre de Santé et Promotion Sociale (CSPS) was located within the community, and iii) it was locally confirmed that the area was safe.

### Sampling of participants

Data collection involved semi-structured interviews (participants = 20) and focus groups (participants = 15). Three categories of participants were recruited: Burkinabe mothers, their husbands, and representatives of local women’s organizations as key informants. These three categories of participants enhanced the representativeness of perspectives regarding the themes under study. Participants were recruited using intentional sampling until data saturation was achieved. Data saturation was reached as soon as the new material produced no change to the codebook [22]. The perspectives of different participants were triangulated, as well as different data collection methods, to ensure that data saturation was achieved.

*Semi-structured interviews*. In each community, a representative of the local women’s organization was first recruited as a key informant with the assistance of the health personnel of the CSPS. Interviews with these individuals allowed for a better understanding of how women’s decision-making power was perceived in the community. Later, two women of reproductive age (between 18 and 49) who have already had at least one child were recruited in each community. All mothers (n = 8) who participated were from different households. They were recruited either at the CSPS during routine consultations or directly from their homes with the help of a community-based health worker who was present at the time of recruitment. After the interview with each mother, her spouse was also approached and recruited for the study. All spouses agreed to participate (n = 8).

*Focus groups.* Two focus groups were conducted: one with mothers aged 18 to 30 (n = 6), and another with mothers aged 30 to 49 (n = 9). Focus group participants were from the communities of Batondo and Tiékouna. Recruitment was convenient and took place at the CSPS, where women were gathered for an infant group visit.

### Data collection

Data collection was carried out in late January 2020. Interviews were conducted by a Burkinabe research assistant sociologist, trained in the collection of qualitative data and able to speak in Moore and Dioula, the most common languages in the study area. The same researcher also moderated the focus group discussions, whose arrangement was facilitated by a community-based health worker. Data collection took place either at the home of the participants (interviews with mothers and their spouses) or at health centers (interviews with key informants and focus group discussions). An interview guide for each category of participant was developed, pre-tested and validated before being used for data collection. The pre-test consisted of a serie of validations with collaborators of the research team. Interviews with key informants were used to refine guides or suggest probing questions. Both interviews and focus group discussions were recorded by the interviewer. Themes and questions addressed during the interviews and group discussions are available (see Table 1). The full interview and focus group guides are available in English in Additional file 1.

**Table 1:** Main themes discussed during data collection

Theme	Example of question
Women’s agency in general	How are decisions made within the household in general? How are you involved in those decisions?
Women’s agency in matters of health	
Decision-making process in matters of reproductive health	How are decisions about your reproductive health made? For example, about antenatal and postnatal visits, or for delivery?
Decision-making process in matters of family planning	How is your family-in-law involved in the decision to use contraception?
Decision-making process in matters of children care	When decisions have to be made about your children (health, education, etc.), how are these decisions made?
Factors influencing women’s agency in reproductive healthcare	
The user fee exemption policy	In your opinion, has the user fee exemption policy influenced the way decisions are made in your household? If so, can you tell us what has changed?

### Conceptual framework

In this study, women’s decision-making power was based on Naila Kabeer’s [23] conceptual framework on empowerment. Kabeer defines power as “the ability to make choices” [23], and argues that empowerment is a process “by which those who have been denied the ability to make choices acquire such an ability” [23]. Empowerment entails change, and is comprised of three dimensions: agency, resources, and achievements. Agency is defined as a person’s ability to set goals and take actions to reach them. Decision-making power, along with participation, is embedded within the concept of agency and refer specifically to the role of women in production of decision.

Due to the cross-sectional nature of this study, it was not possible to investigate empowerment per se, which is a process. Despite its limitations, the decision was made to focus on women’s decision-making power, which has been acknowledged in many studies as a reliable indicator of women empowerment [24, 25]. However, Kabeer’s framework is relevant since our intention is to investigate decision-making power as a key component of women’s agency and, ultimately, empowerment.

### Analysis

Analysis of qualitative data was carried out according to the principle of thematic analysis [26]. The interviews and focus group discussions were recorded during data collection, transcribed verbatim by research assistants and translated into French. The translation was made by members of the research team fluent in both languages (French and the interview language). The translated material was validated by the field researcher, who compared the French translations to the audio recording.

Data was organized and the verbatim transcriptions were read several times. A vertical reading was done to become more familiar with the content of the data, followed by several horizontal readings (sentence by sentence) to begin to identify the first ideas. Categories were developed from participants’ speech and used to codify the verbatim. This was an open, inductive coding to remain very close to the data collected. Codes are available in Additional file 2. Finally, data was interpreted to bring out and clarify the meaning for the readers. Codes were cross-referenced with participant characteristics to enrich the analysis, and Kabeer’s conceptual framework on empowerment was used to guide the interpretation of the results.

### Ethics

The research project has been approved by the University of Montreal’s Research Ethics Committee (certificate #108553) and by the Ethics Committee for Health Research in Burkina Faso (certificate #2018–6-075).

All the participants were solicited and recruited verbally. Participation in our study was not remunerated. The voluntary, free and informed consent of each participant was obtained in writing, using information and consent forms. Individual interviews were conducted in a location where the participants were isolated, so that they could express themselves freely and in confidentiality.

## Results

Table 2 presents the main sociodemographic characteristics of the participants. A total of 20 participants were recruited for the semi-guided interviews and 15 for the focus groups. As is often the case in Burkina Faso, almost all of the participants in our study lived with extended family, usually with the parents or brothers of the husband. Almost half of the participants live in polygamous marriages. The vast majority of women who participated in the study never attended school, but most of them were involved in an income-generating activity, such as manufacturing and selling “dolo” (traditional beer) or agriculture. Women who have had more than three children are the most represented in the study.

**Table 2:** Participants’ socio-demographic characteristics

	Women (n = 23)	Husbands (n = 8)	Key informants (n = 4)
Age			
18–24	7	1	0
25–34	8	3	0
35 and older	8	4	4
Marital status			
Married (monogamous)	13	5	2
Married (polygamous)	10	3	2
Ethnicity			
Gourounsi	9	4	2
Karaboro	9	2	1
Turka	2	2	1
Dagara	2	0	0
Mossi	1	0	0
Education			
Never attended school	11	1	0
Primary school	5	3	2
Secondary school	7	2	2
Koranic school	0	2	0
Number of children			
1	3	2	0
2	3	1	0
3	6	3	0
More than 3	11	2	4
Income generating activity			
Yes	14	8	4
No (in school)	3	0	0
No (housewife)	6	0	0
Household composition			0
Nuclear family	2	0	0
Extended family	21	8	4

### Power structures within households

Most of the participants described good family relationships, both within the couple and among the other members of the household. From the perspective of the husbands, conflicts can arise when a spouse is judged too independent and does not follow the directive of her husband. Such issues did not emerge from the women’s perspective.

“[The family relations] *go well. Except with one of the women, the last one. She wants to wear the pants; so much so that this year I did not allow her to work with me in the garden*. […] *Often, she doesn’t even inform me, she makes her decisions alone if she wants to do something. This is what makes us not get along. The other women follow my decisions, the last one is the problem*.” (Husband #1, CSPS B)

Social norms and the status of women often shift the way decisions are made in Burkinabe families. In rural Burkina Faso, social and cultural norms usually ensure that women rely on the authority of their husbands, who are presumed to be the head of the household and held responsible for taking care of all its members. Moreover, it is common for different households to live in a shared yard. Living in a large concession could further limit women’s decision-making power, as members of other households could pass judgment on a couple in which the woman makes decisions without her husband’s approval.

“*So that’s why I said that the ones* [the women] *who can decide are not many. Because in a concession, if you, the woman, you can decide, people say that you grabbed your man there in your hand* [the woman commands her husband] […] *And if it was like, everyone is at home, then it would be fine*. *In a common yard* […] *even if you are going to decide, it would be in secret but not in front of people.* […] *It creates a lot of problems.*” (Key-informant, CSPS A)

Although the wife is dependent on the authority of her husband, most of the time she must also refer to her husband’s parents, and/or to her husband’s older brothers. Because they are older, they often hold a greater power regarding household decisions. When there is a decision to be made, no one should undertake a project without consulting them, not even the husband.

“*You are not alone; so, you can’t tell the woman to go* [to do something] *without taking other family members into account. In the family there are people who exceed me in age, we must listen to them too*” (Husband #1, CSPS A)

Similarly, when a woman has one or more co-wives, the order of arrival of the wives in the household also affects the decision-making process. The more recently the woman arrived into the household, the less decision-making power she has. The co-wives can sometimes give their point of view on the decision to be taken. However, their husband will not always consider their opinion, particularly if it goes against the decision he makes.

“*As I am the third* [wife], *if the first two* [wives] *do not find fault, it is not up to me to say something. When we feel that his decision is well-founded, we support him. Otherwise we can tell him what we think even if he will not take into account what women will say*”. (Women #1, CSPS B)

### Women’s autonomy in decision-making

**Fig. 2:**
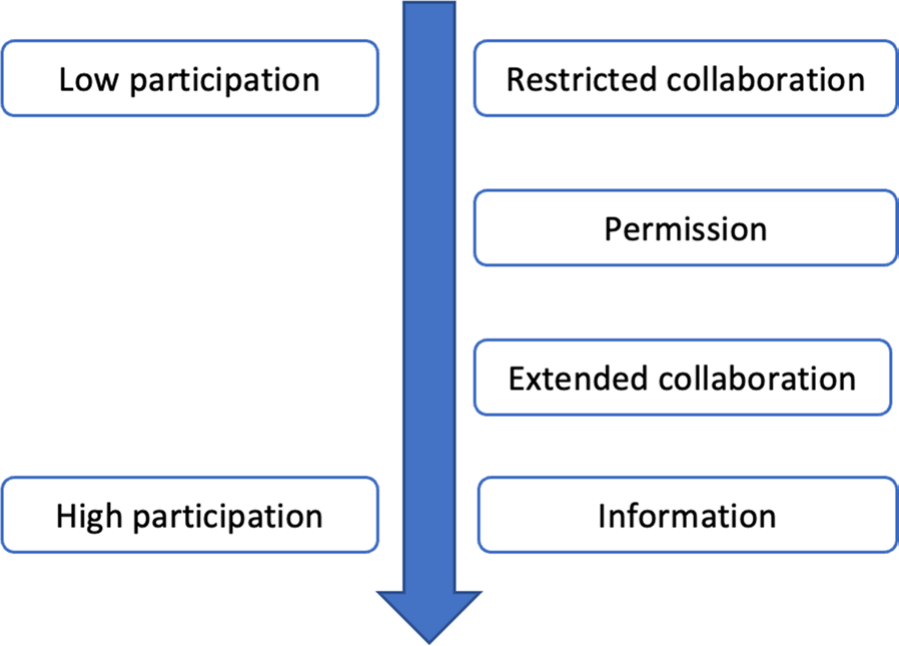
Continuum of women’s decision-making power

The analysis highlighted different decision-making processes regarding everyday life and healthcare seeking within households. Figure 2 provides a representation of these different processes according to the degree of women’s participation. Four types of decision-making processes are presented below: information, extended collaboration, permission, and restricted collaboration. It is noteworthy that these processes are not entirely distinct, sometimes they overlap in the decision-making process. Also, each process does not strictly correspond to a particular type of decision, and therefore the categorization should not give the impression of pre-determined scenarios regarding the decision-making process. However, the analysis revealed specificities in the decision-making process regarding access to family planning, therefore, this topic will be discussed in a separate section.

### Information

In certain circumstances, it is possible to only inform one family member before visiting the health center for childcare, without formally requesting permission to go. Women should ideally inform someone before bringing the child to the hospital, but in the absence of the husband or other members of the household, women can go to the CSPS on their own initiative, thanks to the user fee exemption policy for children under five.

“*She can go and treat him* [the child], *she can say, ‘the child was sick, I did not see [the mother of the husband], I did not see the brothers, you too were not there, but I went* [to the health center] *I was given the medicine, he is taking the medicine’*. […] *If everyone is there, she asks and she is told to go* [to the health center]; *if people are not there, she can go and take care* [of the child], *as it’s free there.*” (Husband #1, CSPS A)

Regarding reproductive healthcare, it is important for women to inform a family member, but only for safety reasons, so as not to worry relatives when they are away and to avoid family conflicts when they return.

“*Because when I go out without informing them, they won’t know where I went, when I get back, they can fight with me, they won’t know I was gone* [to the health center], *maybe you can have a problem along the way. For that, you have to inform them that you want to go somewhere and then come back.*” (Woman #2, CSPS A)

For most participants, other household members, and in particular the husband’s mother, are notified at the time of delivery but do not intervene in the decision-making process regarding whether or not the women should deliver in a healthcare facility. Rather, it is to inform them so that the woman who gives birth can count on the support of her in-laws at the CSPS.

“*My parents are involved in childbirth. Especially my mother because she is the one who accompanies my wife to the maternity unit. She is the one who washes the baby for the first few days and takes care of my wife while waiting for her to regain a little more strength.*” (Husband #2, CSPS A)

### Extended collaboration

When decisions concern all family members, for example when something is to be sold or purchased, decisions are most often made according to a process that could be described as collaboration. Family members get together to discuss and debate the project. For some households, a process of extended collaboration exists, in which women get to participate and give their point of view, but the final decision will be made by the husband, his parents, or older brothers. In this kind of decision-making process, women may be allowed to raise their voice, but from their perspective, it is unclear how much their opinion will be heard.

“*It is my husband who decides that we are going to put the children in school, he talks to his brothers, his brothers also say that there is no problem, that he can put them in school. It is the husband who makes the decision for everything and we follow what he said. But if it is a bad decision, we can tell him that it is not good, he can do as he pleases.*” (Woman #2, CSPS D)

### Permission

When the decision to be made concerns women only (for example, if a woman wishes to exercise an income-generating activity or to visit her parents), she must obtain authorization to do so. Most often she will address her husband. Sometimes she will also need to obtain permission from her in-laws; however, the authorization will usually be granted if the project is considered suitable. However, the permission being granted varies greatly according to the husband’s opinion of the project the woman wants to undertake.

“*If she has to do it* [any activity the woman wants to do], *her husband decides, but a woman cannot go to an activity if her husband does not know about it. It is her husband who must authorize her to go and do her business.* […] *But if he sees that the activity can be beneficial for him too, he cannot refuse.*” (Husband #1, CSPS C)

When a woman is pregnant and feels she needs seek care at a health center as part of her pregnancy, she will usually take the initiative to talk to her husband about it. His authorization will be necessary for her to move forward in seeking healthcare; however, in matters of health, permission is usually granted. This includes decisions concerning access to healthcare for children.

“*If the woman is pregnant, she must know it herself, but if she sees that her pregnancy has reached like two or three months, she must come to the hospital but before coming she must speak with her husband. If her husband agrees, he will accompany her to find out how it will go over there. If* [the question] *is to go to the hospital because of illness, no one can refuse.*” (Husband #1, CSPS C)

### Restricted collaboration

Finally, in other households, when there is a decision to be made that is related to work but affects all family members, such as plans for agriculture work, the husband often organizes a meeting with his parents and his older brothers. Women are not allowed in these meetings and are therefore excluded from the decision-making process. This decision-making process can be defined as restricted collaboration, a phenomenon that was mentioned most often by male participants.

“*We have a lot of things we do at home, but if we have to do these things, we need to have a meeting, so everyone is informed. For example, if it is to go and sell something to solve a problem, we have to agree because it is our big brother who makes the decision. If we all agree, he can do it, but if some disagree, we will get along*. […] *The woman has nothing to do there* [in those meetings]. *It is the decision of the men in the yard*.” (Husband #1, CSPS C)

### Decision-making process for family planning

Almost unanimously, participants testified that in the case of contraception, the authorization of the husband is still necessary, despite the recently implemented user fee exemption policy for family planning services in the regions of our study. Unlike maternal and child healthcare, women cannot simply inform their husbands before seeking family planning services. A woman is likely to be stigmatized if she uses contraceptive methods without having obtained her husband’s agreement, because members of her community would perceive her as a woman who is unfaithful to her husband.

“*On the other hand, I think that free healthcare has not really had an impact on decision-making. Despite the fact that it is free, it is always men who decide whether women should do family planning or not. If you made the decision without informing your husband, you are called a disrespectful woman.*” (Woman #3, focus group at CSPS A)

According to most of the participants, a specificity of family planning decisions is that women’s parents-in-laws are often excluded from the decision-making process. Because several misconceptions about family planning are persistent, the family-in-law may forbid or try to dissuade the couple from using contraception, which is why the family-in-law is not included in the discussions regarding family planning. Specifically, the in-laws might fear the risk of infertility for women, or even that women could have extramarital relationships.

“*They* [women’s parents-in-law] *don’t agree because they think that when you go to do family planning, it’s to look for the boys or you don’t even want to give birth anymore. That on top of that, if you do family planning* […] *that can lead you to be sterile, you will no longer give birth. That is why they do not want to accept that their sons’ wives go to do family planning*.” (Woman #2, CSPS A)

### Changes in women’s decision-making power

According to some of our participants, the introduction of the user fee exemption policy may have changed the way decisions are made within households regarding access to healthcare. Indeed, now that it is provided free of charge, women no longer need formal authorization from their husbands (nor from their in-laws) before seeking healthcare. Rather, they can simply inform them. This perspective came mostly from women participants.

“*Now that it is free, whether it is the child or the man who is sick, or the woman who is sick, you only have to inform someone in the yard that you are sick and that you want to go to the hospital, and you come to treat yourself, that’s all.*” (Key-informant, CSPS B)

This change does not apply to everyone. In some households, the decision-making process has remained basically the same. Whether treatment is free or not, women still need their husband’s permission before going to a health center. However, the change lies in the fact that the authorization to visit the health center should now always be granted by the husband, thanks to the introduction of the user fee exemption policy. A family’s lack of financial resources can no longer be used by husbands as a reason to forbid their wives from seeking reproductive healthcare and family planning services.

“*It is always the same thing but as it is health, nobody can object, especially since healthcare is free, you do not ask for money.*” (Woman #3, focus group discussion at CSPS A)

“*Free family planning really has an impact on decision-making. Before, when it was profitable, men hid behind the question of money to justify their refusals to allow their wives to use contraceptive methods, but with free healthcare, they have no more pretext.*” (Woman #1, focus group discussion at CSPS A)

From the perspective of most of the male participants, the user fee exemption policy did not change the decision-making process, nor did it give more decision-making power to their wives. Most of them mentioned the “permission” process regarding access to healthcare, as if their authorization is always mandatory, whether healthcare is free or not.

“*No, free healthcare has had no effect on decision-making. In any case, whether healthcare is free or not, I make the decisions concerning her reproductive health or the health of her children, especially since not all products are covered by free care and that at times I have to pay for certain products.*” (Husband #2, CSPS A)

## Discussion

This study proposes a conceptual framework describing women’s decision-making power within their household, based on a continuum ranging from no participation to strong participation. Women’s participation in the decision-making process is variable and depends on several factors, including the type of decision to be made, the type of healthcare services to be sought, her age and rank in the household, and social norms in the community. Regarding access to reproductive healthcare services, women do not have full decision-making autonomy, even in a context where direct payment has been abolished. On the other hand, the user fee exemption policy may have facilitated the negotiation process within the household, therefore making access to healthcare easier.

With the framework proposed in this study, the aim was to contribute to conceptualize women’s decision-making power, both in terms of day-to-day decisions and access to reproductive healthcare, childcare, and family planning. Other studies have measured women’s autonomy regarding access to healthcare in low- and middle-income countries. A study conducted in Ethiopia also suggests that women’s autonomy may be defined by her participation in domestic decision-making and her ability to seek permission to obtain medical care [27], but to our knowledge, this study is the first to propose a conceptual framework such as this in a context of free healthcare in Burkina Faso [28]. Another innovative component of this framework is that it proposes to categorize the decision-making processes according to the importance given to women’s participation in decision-making by household members.

However, this model has limitations. For example, other components of decision-making power could have been examined, such as women’s freedom of movement or whether they have the final say on various other matters, such as what to cook, children’s clothes, or visiting relatives [29]. Moreover, the data do not indicate precisely how these different decision-making processes take place or how household members interact from the initiation of a project to the final decision. As suggested in a study in Nepal, “interspousal communication” can be another dimension of women’s autonomy [30]. To better describe and categorize the collaborative process, it may have been useful to examine the degree of women’s participation in the collaborative decision-making process itself, and the extent to which women are able to make their voices heard.

Finally, this model does not describe the social and structural determinants that may influence women’s decision-making power, such as inequalities based on gender and socioeconomic and political contexts that can stratify power relations within societies [31]. Similarly to what has been observed in other countries, women’s empowerment and decision-making power in Burkina Faso is correlated with household characteristics, such as the socioeconomic status and education level [32]. Moreover, women living in urban areas of Burkina Faso tend to participate more in the decision-making process than women living in rural areas [10]. Women’s decision-making power does not evolve independently of other structural factors, all of them which need to be addressed to enhance women’s position in the Burkinabè society.

Results show that, by facilitating negotiation processes with members of their households, the national user fee exemption policy seems to have contributed to improving access to reproductive healthcare. In fact, while the spouse’s authorization is still required before visiting a health center for reproductive healthcare, our results showed that this authorization would be systematically granted. As the model shows, in some households, women need only inform their husbands or in-laws before joining a healthcare facility rather than waiting for approval [18]. This is an improvement over before the policy was introduced in Burkina Faso, because when healthcare was not free of charge, women were forced to engage in negotiation processes to obtain the money to pay for medical care [33]. A similar result has been shown in Mali, where the fee-for-service system has reduced women’s agency regarding access to reproductive healthcare for themselves and malaria treatment for their children [34]. Other studies have examined the effects of other fee abolition policies on women’s decision-making power, especially in education. For example, women who have been exposed to a tuition-free primary education had a greater chance of having some say in decisions about their health [35], marriage decisions, and fertility [36]. In Brazil, a free vocational training program has helped provide women with income and employment, which has also given them a greater sense of their civil rights [37].

Despite the user fee exemption policy for family planning, women’s decision-making power regarding access to contraception seems to be lower than their decision-making power for obtaining other reproductive healthcare. Studies show that when a society is built on a patriarchal model, the husband will often be the sole decision maker with respect to contraception, and women must comply with their husband’s family planning decisions [38]. Medical anthropological studies have described how men can have a strong influence on contraception, pregnancy, childbirth, and abortion [39]. Thus, women generally remain dependent on the permission of their husbands regarding family planning, so strategies to improve access to family planning should include awareness targeting men. It has been shown that contraceptive methods are more likely to be used when the decision comes from the couple and not the women only [40]. In Malawi, for example, an educational intervention around family planning targeting men has been shown to be effective in improving communication between spouses around family planning issues, which in turn facilitates consensus between a man and his wife [41]. Another intervention could be promoting the female condom, which has proven to be an efficient method to avoid unwanted pregnancy as well as a tool for empowering women, in that they have greater ability to negotiate safer sex with men [42].

This study is built on Naila Kabeer’s conceptual framework on empowerment [23]. The framework suggests that empowerment comprises three components: resources, agency, and achievements. According to Kabeer, agency can be described as decision-making, participation and negotiation. With decision-making power being the main focus of the study, we acknowledge that it is strongly connected to empowerment. Thus, improving women’s decision-making power and participation within their households might result in greater empowerment.

In addition to reproductive health and family planning, numerous studies have shown the benefits of women’s empowerment on their health in a broader sense. For example, in India, women with access to a bank account and a mobile phone are more likely to have access to menstrual hygiene products [43]. Empowering women can reduce their risk of contracting HIV/AIDS, and economic empowerment can make it less difficult for women to cope with the virus [44]. In India and Nigeria, women who are empowered are more likely to have healthier children [45]. Thus, in order to achieve the Sustainable Development Goals by 2030, women’s empowerment and gender inequalities are essential elements to consider during the implementation of public health interventions, especially in low- and middle-income countries.

A particularity of this study is that the decision-making process was also examined from the point of view of the women’s husband. Results showed some differences in their perspective, as the husbands tend to emphasize the importance of their permission in the decision-making processes, while women were more inclined to only inform their husband whenever possible (for visiting a healthcare facility, for example). Moreover, men tend to perceive women who decide by themselves as too independent, resulting in family conflicts. As highlighted in a recent study, contrasting perspectives in the couple can have ambivalent effects on women. Indeed, women taking power by themselves instead of waiting for their husband to give it to them may gain better health and well-being outcomes [24]; however, intimate partner violence and emotional violence may also arise [24]. Interventions to promote women’s decision-making power and targeting men may help women gaining more power without being at risk of gender-based violence.

### Limitations

This study was cross-sectional and took place in two rural settings in Burkina Faso, which limits the transferability of the results. These settings were part of the two pilot regions where user fees for family planning were removed; results may differ from what would have been observed in a setting with such a policy implemented after under routine conditions. A Hawthorne effect cannot be ruled out, and it is possible that a social desirability bias has affected the participants’ answers on sensitive topics [46]. Since most of the participants were recruited at their homes, some may have felt uncomfortable discussing gender-based norms and power imbalance. Several measures were taken to limit such bias, notably the use of a surveyor experienced in qualitative research about sensitive issues, the conduct of interviews in places that ensured confidentiality, and triangulation of information obtained from different sources. In focus group discussions, participants were gathered with individuals of the same sex and age category to further reduce the risk of information bias.

## Conclusion

This study shows that in a context of free reproductive care and family planning in Burkina Faso, women’s lack of autonomy in decision-making constitutes a persistent barrier in healthcare access. Women remain dependent on the permission of their husbands (and sometimes their in-laws), especially for access to family planning. While user fee removal policies alleviate the financial barrier, they do little to empower women by challenging the decision-making process in the household. Interventions targeting women’s agency and empowerment are required to prevent further health inequities. Other interventions that involve husbands and community members are also necessary to address social norms, as they are important determinants of women’s empowerment.

## Supplementary Information

**Additional file 1.** Interviews and focus groups guides.

**Additional file 2.** Codes.

## Data Availability

The datasets used and/or analyzed during the current study are available from the corresponding author on reasonable request.

## Abbreviations

CSPS: Centre de santé et de promotion sociale (Health and Social Promotion Center)
UN: United Nations
WHO: World Health Organization
