# Supplementary material for: Women’s decision-making power in a context of free reproductive healthcare and family planning in rural Burkina Faso
Source: BMC Womens Health. 2021 Jul 22;21:272. doi: 10.1186/s12905-021-01411-4 (PMC8296726; doi:10.1186/s12905-021-01411-4)
Supplement: Supplementary file 1 — Additional file 1. Interviews and focus groups guides. [file 12905_2021_1411_MOESM1_ESM.docx]

**Women’s decision-making power in a context of free reproductive healthcare and family planning in rural Burkina Faso**

Beaujoin, C., Bila, A., Bicaba, F., Plouffe, V., Bicaba, A., Druetz, T.

Additional File 1 – Interviews and focus groups guides

**INTERVIEW GUIDE - WOMEN**

Socio-demographic questionnaire

- Age
- Ethnic origin
- Marital status
- Household composition
- Number of children
- Level of education
- Economic activity

A) General Decision-Making Reports

1. Can you tell us about your relationship with your husband in the household? How do you think this relationship has changed?

2. How are decisions made in the household in general? How do you participate in them?

3. When decisions that affect you need to be made, what role does your husband and/or other family members play in the decision-making process?

4. When decisions need to be made about your children (health, education, etc.), how are these decisions made?

B) Reproductive and maternal health

5. When you think you need reproductive health care (prenatal, postnatal, delivery) who do you go to and why? What role does your family play in this decision-making process regarding such care?

6. How are decisions about your reproductive health made? For example, about prenatal, postpartum, or delivery visits? Can you tell us about decision-making about your delivery?

7. In your opinion, has free maternal and child health care affected the way decisions are made in your household? If so, can you tell us about what has changed? Has the change been in maternal care? Childcare? Both?

C) Family planning

8. How are decisions about your family planning plans in the household made? (Desire to have children, birth spacing, use of contraception...)

9. Have you ever visited a health center to receive contraception? How was this decision made? Were other family members involved in making the decision?

10. Would you like to see family planning made free in your area? Why or why not? How would it change for you if family planning were free? And for women in general?

D) Decision-making autonomy in general

11. Do you feel that the way you make decisions has changed in your household? If so, in what way and for what reason?

12. What change would you like to see to bring about more decision-making autonomy for women in general?

13. Do you have anything else to add?

**INTERVIEW GUIDE - HUSBANDS**

Socio-demographic questionnaire

- Age
- Ethnic origin
- Marital status
- Household composition
- Number of children
- Level of education
- Economic activity

A) General Decision-Making Reports

1. Can you tell us about your relationship with your wife in the household? How do you think this relationship has changed?

2. How are decisions in the household generally made? How do you participate in them?

3. When decisions that affect your wife need to be made, how are you involved?

4. When decisions need to be made about your children (health, education...), how are these decisions made?

B) Reproductive and Maternal Health

5. How are decisions about your wife's reproductive health made? For example, with regard to prenatal, postnatal, or delivery visits?

6. When your wife thinks she needs reproductive health care (prenatal, postpartum, or delivery visits), who does she go to and why? What role do you and other members of your household play in this decision-making process?

7. In your opinion, has free maternal and child health care affected the way decisions are made in your household? If so, can you explain what has changed? Is the change in maternal care? Childcare? Both?

C) Family Planning

8. How are decisions about family planning projects in the household made? (Desire to have children, birth spacing, contraception...)

9. Has your wife ever visited a health center to receive contraception? How was this decision made? Which family members were involved in making this decision?

10. Would you like to see family planning become free in your area? Why or why not? What do you think would change for your wife if family planning were free? And for women in general?

D) Decision-making autonomy in general

11. Do you feel that the way you make decisions has changed in your household? If so, in what way and why?

12. Would you like women to have more decision-making autonomy in general? If so, what would need to change to achieve this?

13. Do you have anything else to add?

**INTERVIEW GUIDE - WOMEN'S ORGANIZATION REPRESENTATIVES**

Socio-demographic questionnaire

- Age
- Ethnic origin
- Marital status
- Household composition
- Number of children
- Level of education
- Name of organization
- Role in organization

1. Can you tell us about your organization (activities carried out, date of creation...)? How many members does your organization have?

A) General decision-making relationships

2. How would you describe the relationships and family life of women in your community in general?

3. How do you perceive the ability of women in your community to make decisions in general?

4. In your opinion, how are decisions concerning children (health, education, etc.) made within the household?

B) Maternal and reproductive health

5. Can you tell us about the experiences (provider-patient relationship, appreciation of care, personal experiences) that women in your community have regarding reproductive health in health centers? For example, prenatal, postnatal, or delivery visits.

6. How do you think decisions about women's reproductive health (prenatal, postnatal or delivery visits) are made in the household?

7. Do you think that free maternal and child health care has had an influence on the way decisions are made in the household? If so, what do you think has changed?

C) Family planning

8. How are decisions about family planning projects made in the household? (Desire to have children, birth spacing, contraception...)

9. Do you know if women in your community use family planning services to receive contraception? Do you know how this decision is made within the household? How are other family members involved in these decisions?

10. Would you like to see family planning made free in your area? Why or why not? What do you think women in your community think about this? How would it change for women in your community if family planning were free?

D) Decision-making autonomy in general

11. Do you feel that the decision-making process has changed within households in your community? If so, in what way and why?

12. Do you feel that your organization has empowered women? If so, in what way?

13. Would you like women to have more decision-making autonomy in general? If so, what would need to change to achieve this?

14. Do you have anything else to add?

**FOCUS GROUP GUIDE**

Socio-demographic questionnaire

- Age
- Ethnic origin
- Marital status
- Household composition
- Number of children
- Level of education
- Economic activity

Decision-making power

- Can you tell us about your relationship with your husband in the household?
- How do you feel about couple relationships in your community in general?
- How do you participate in making decisions that affect the whole household?
- How are decisions that affect you made within the household?
- How are decisions about your reproductive health care (prenatal, postpartum, and delivery) made?
- How are decisions about your children made within the household?
- What about your family planning plans (birth spacing, contraception, etc.)? Would you like to see family planning services become free in your area? Why or why not? What would it change for you and for women in general?
- Do you think that free maternal and child health care has influenced your decision-making power? If so, can you tell us what has changed?
- Do you participate in women's community activities? If yes, how has this influenced the way you make decisions? If no, would you like to do so?
- Would you like to see women have more decision-making autonomy in general? What changes do you think should be made to achieve this?
