# Supplementary material for: Women’s decision-making power in a context of free reproductive healthcare and family planning in rural Burkina Faso
Source: BMC Womens Health. 2021 Jul 22;21:272. doi: 10.1186/s12905-021-01411-4 (PMC8296726; doi:10.1186/s12905-021-01411-4)
Supplement: Supplementary file 2 — Additional file 2. Codes. [file 12905_2021_1411_MOESM2_ESM.docx]

Additional file 2: Codes

1. **Maternal and child health**

- **Free healthcare**
  - Benefits of free healthcare
  - Problems caused by free healthcare
  - Changes observed with free healthcare
  - No change with free healthcare
- **Family planning**
- Social acceptance of family planning
- Benefits of family planning
- Family conflicts
- Religious beliefs
- Family planning awareness
- Stigma of family planning
- **Child health**
  - Household involvement in child health
  - Compliance with childcare
- **Maternal health**
  - Barriers to accessing maternal care
  - Compliance with maternal care
  - Visiting patterns
  - Maternal Challenges
  - Household Involvement in Maternal Health
  - Awareness of access to maternal care

1. **Family Relationships**

- **Family relationships**
  - Good family relations
  - Poor family relationships
  - Changes in family relationships
  - No change in family relationships
- **Family hierarchy**
  - Respect for family hierarchy

1. **Decision-making process**

- **Decision-making process in the everyday life**
  - Authorization
  - Collaboration
  - Exclusion
  - Information
- **Decision-making about women’s project**
  - Authorization
  - Collaboration
- **Decision-making about the children**
  - Authorization
  - Collaboration
  - Information
- **Decision-making about reproductive care**
  - Authorization
  - Collaboration
  - Exclusion
  - Information
- **Decision making about family planning**
  - Authorization
  - Collaboration
  - Exclusion
  - Information
- **Factors that support women’s decision-making power**
  - Acceptance by men
  - Other
  - Family collaboration
  - Education
  - Women’s groups
  - Awareness
  - Financial support
  - Women’s work and activities
  - Travel
- **Factors limiting women’s decision-making power**
  - Ethnicity
  - Opposition from men
  - Family and community life
- **Changes within the household**
  - Change in decision-making
  - No change in decision making

1. **Other codes**

**Financial and material difficulties**

**Solidarity**
